# A RADAR-Based Assay to Isolate Covalent DNA Complexes in Bacteria

**DOI:** 10.3390/antibiotics8010017

**Published:** 2019-02-27

**Authors:** Katie J. Aldred, Adeline Payne, Olivia Voegerl

**Affiliations:** Biology Department, University of Evansville, Evansville, IN 47722, USA; ap280@evansville.edu (A.P.); ov4@evansville.edu (O.V.)

**Keywords:** Quinolone, topoisomerase, gyrase, RADAR assay, ICE assay, covalent complex

## Abstract

Quinolone antibacterials target the type II topoisomerases gyrase and topoisomerase IV and kill bacterial cells by converting these essential enzymes into cellular poisons. Although much is known regarding the interactions between these drugs and enzymes in purified systems, much less is known regarding their interactions in the cellular context due to the lack of a widely accessible assay that does not require expensive, specialized equipment. Thus, we developed an assay, based on the “rapid approach to DNA adduct recovery,” or RADAR, assay that is used with cultured human cells, to measure cleavage complex levels induced by treating bacterial cultures with the quinolone ciprofloxacin. Many chemical and mechanical lysis conditions and DNA precipitation conditions were tested, and the method involving sonication in denaturing conditions followed by precipitation of DNA via addition of a half volume of ethanol provided the most consistent results. This assay can be used to complement results obtained with purified enzymes to expand our understanding of quinolone mechanism of action and to test the activity of newly developed topoisomerase-targeted compounds. In addition, the bacterial RADAR assay can be used in other contexts, as any proteins covalently complexed to DNA should be trapped on and isolated with the DNA, allowing them to then be quantified.

## 1. Introduction

DNA topoisomerases are essential, ubiquitous enzymes that maintain the topological integrity of the genome [1,2,3,4,5,6,7,8,9,10,11]. There are two classes of topoisomerases—type I topoisomerases and type II topoisomerases. Although both regulate DNA topology, they differ in the types of topological issues they can resolve due to their differing mechanisms. Type I topoisomerases cleave one strand of the double-helix to relieve torsional stress in the genome [2,7,8,9,10,11]. Meanwhile, type II topoisomerases cleave both strands of the helix to not only relieve torsional stress and maintain the appropriate level of supercoiling, but also to resolve knots and tangles that arise as a result of normal cellular processes and decatenate daughter chromosomes during replication [1,5,6,12,13,14,15,16,17,18,19]. Due to their mechanisms of action that require breaking the DNA backbone, these enzymes are inherently dangerous to the cell.

**Figure 1:**
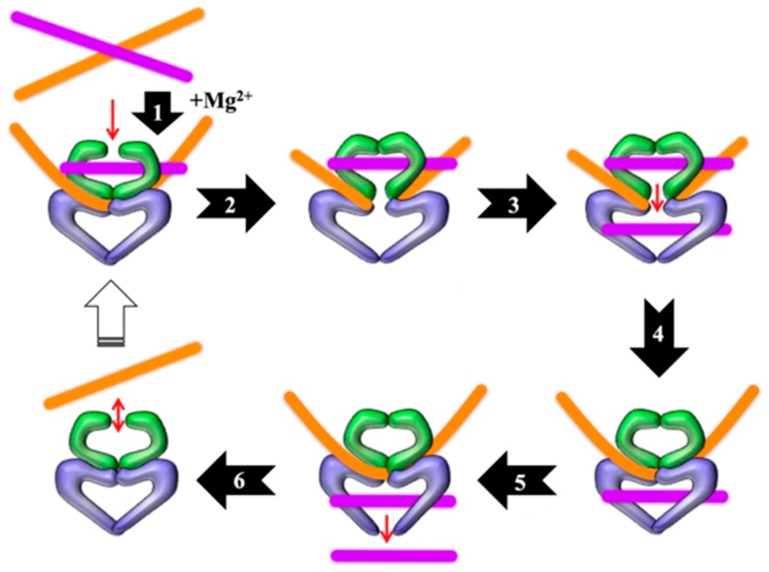
Catalytic cycle of type II topoisomerases. Step 1: The enzyme bends the gate-, or G-, segment of DNA in the presence of divalent metal ions (the physiological ion is Mg^2+^). Step 2: The enzyme cleaves and covalently attaches to the newly generated 5’-termini of the G-segment, generating the cleavage complex. Step 3: The enzyme passes the transfer-, or T-, segment of DNA through the cut it generated in the G-segment. Step 4: The enzyme religates the G-segment. Step 5: The T-segment is released from the enzyme. Step 6: The enzyme releases the G-segment and resets for another round of catalysis. Note that ATP hydrolysis is required for the enzyme to complete the catalytic cycle. Modified from Reference [22].

The bacterial type II topoisomerases, which are named gyrase and topoisomerase IV, are A_2_B_2_ heterotetramers in which the subunits are not covalently bonded to one another [2,5,6,20]. During their catalytic cycles (Figure 1), the enzymes cleave both strands of the DNA helix and covalently attach to the newly generated DNA ends. This covalent attachment occurs between the active site tyrosine residue in the A subunit of the enzyme and the 5’ terminus of one strand of the DNA helix. Thus, each enzyme forms two covalent bonds: one between each A subunit and one or the other of the DNA strands of the helix. Once the enzyme has cut and is covalently attached to the DNA strands, a “cleavage complex” is formed. In the absence of a drug that acts as a topoisomerase “poison,” the integrity of the DNA will be restored by the enzyme during religation [2,3,4,5,6,7,8,9,10,11,20,21].

Both human and bacterial topoisomerases are important drug targets [5,13,20,21,23,24,25,26,27,28,29]. In humans, anticancer drugs, such as etoposide, kill cells by converting the human type II topoisomerases into potent cellular poisons [5,23,24]. Similarly, the quinolone antibacterials target topoisomerase IV and gyrase, the two bacterial type II topoisomerases [13,20,25,26,27,28,29]. The quinolones are a highly prescribed group of broad-spectrum antibacterials [12,14,15,16,17]. Their clinical efficacy, though, is being threatened by increasing levels of resistance, which most often result from specific mutations in gyrase and topoisomerase IV that disrupt the water–metal ion bridge interaction between the drug and enzyme [13,20,29,30,31,32]. Quinolones kill bacteria by intercalating into the topoisomerase-generated break in each DNA strand and thereby prevent the topoisomerases from completing their catalytic cycles and resealing the strand breaks that they created [20,30,33,34]. These stabilized breaks can then be converted into permanent breaks, such as via collisions with DNA tracking systems or other cellular components that disrupt the cleavage complex. Accumulation of these breaks ultimately results in shredding of the genome and cell death [20,25,26,28].

For many years, topoisomerase II-targeted drugs have been studied in purified systems to understand their mechanisms of action against and interactions with their target enzymes. For drugs targeting the human enzymes, a handful of assays exist that allows for measuring the level of cleavage complexes produced in cultured cells as a result of drug treatment; these various assays have been concisely summarized and analyzed by others [35,36]. The most recently developed assay to accomplish this goal has been termed the “rapid approach to DNA adduct recovery,” or RADAR assay [36,37]. It is similar to the “in vivo complex of enzyme” or “ICE” assay [37,38,39], but uses conditions that negate the necessity of an ultracentrifuge, making it a more widely accessible assay. For a long time, no equivalent method for use with bacterial cells existed [35]. The biggest challenge in the development of a bacterial equivalent is due to bacteria having a cell wall. Because human cells have no wall, lysis can easily be achieved via addition of a detergent, which simultaneously traps cleavage complexes. Although some detergents, such as sodium dodecyl sulfate (SDS), can simultaneously lyse bacteria and trap cleavage complexes, SDS is not compatible with the downstream processing steps, including DNA precipitation. 

A few years ago, an adaptation of the ICE assay to allow its use in bacteria was developed [35]. However, being based on the ICE assay, this method required an ultracentrifuge. In addition, the lysis step took a full hour. Therefore, our goal was to develop a RADAR-based assay [36] that would alleviate the need for expensive and specialized equipment, such as an ultracentrifuge, and would also rapidly lyse the bacterial cells while simultaneously trapping cleavage complexes in order to ensure that these complexes did not dissociate before they could be trapped. After testing a number of chemical and mechanical lysis conditions and combinations, as well as DNA precipitation conditions, a bacterial RADAR assay was developed that includes rapid lysis of the bacterial cells and does not require any specialized equipment. This widely accessible assay, which requires small volumes of reagents, will allow the effects of quinolones or other drugs on topoisomerase IV and gyrase to be measured in the cellular context and could also be used to measure activity of other proteins that covalently attach to DNA.

## 2. Results

### 2.1. Technical Considerations

DNA topoisomerase activity is temperature-sensitive. Large increases and decreases in temperature have been shown to cause dissociation of cleavage complexes in vitro, and smaller temperature changes (above or below 37 °C) can increase or decrease the number of cleavage complexes present [40,41,42]. To this point, when developing a bacterial ICE assay, Aedo and Tse-Dinh noted that “lysis at 37 °C was crucial for good yield of covalent complex and that lysis at 4 °C or 0 °C yielded negative results” [35]. Thus, one consideration when developing this assay was to minimize exposure of the bacteria to temperature fluctuations during growth and drug treatment until cell lysis, at which point the cleavage complexes would be trapped. For this reason, when possible, all steps were performed at 37 °C and all reagents and vessels were warmed to 37 °C before use. To this point, both chemical and mechanical methods of cell lysis were tested, with the mechanical method consisting of five sonication passes. Sonication is well-known to increase the temperature of the subject solution. Thus, when undergoing sonication, the cultures were placed in a 37 °C waterbath between passes to maintain them at a constant temperature. In early development, the temperature of the cultures was measured immediately before and after each pass and was found to not increase by more than 2 °C in this time span. In addition, after cycling through each culture to get to the next sonication pass, the temperature of the cultures had decreased back to ≈37 °C, such that at the end of the five passes, no culture was at greater than 40 °C.

Cleavage complexes formed by topoisomerases in vitro have been shown to have varying stabilities [37,40,43]. For example, *Escherichia coli* topoisomerase IV cleavage complexes with ciprofloxacin have a half-life of less than 5 minutes [41]. Thus, a second consideration for cell lysis and cleavage complex trapping was to make this step as rapid as possible to prevent cleavage complex dissociation during lysis and prior to trapping. M buffer (and its modified version) alone provides such conditions for mammalian cells which lack a cell wall and so can be lysed via addition of a detergent that serves the dual purpose of also trapping cleavage complexes [36,37]. For bacteria that have a cell wall, it was therefore necessary to either lyse the cells using chemical denaturation (which would simultaneously trap cleavage complexes) or mechanically in the presence of a chemical and/or detergent that would trap the cleavage complexes immediately upon lysis.

### 2.2. Early Development

During the initial stages of developing a bacterial version of the RADAR assay, a number of cell lysis and DNA extraction methods were tested, such that a total of 19 different versions of the assay were examined. These methods were compared by blotting the acquired DNA for subunit A of topoisomerase IV and/or gyrase as this subunit covalently attaches to the DNA termini generated by the enzymes during their catalytic cycles. Based on previous studies [33,35,41], it was expected that treating cultures with increasing concentrations of ciprofloxacin would result in an increase in cleavage complexes on the DNA, and thus, an increase in topoisomerases in the blotted DNA fraction. In general, methods were dismissed based on inconsistent results and/or very low DNA yields. Unsurprisingly, the two best methods (16 and 19) were those that were most similar to the original RADAR assay [36,37] that is used with mammalian cells.

### 2.3. Bacterial RADAR Assay Method 16 versus 19

**Figure 2:**
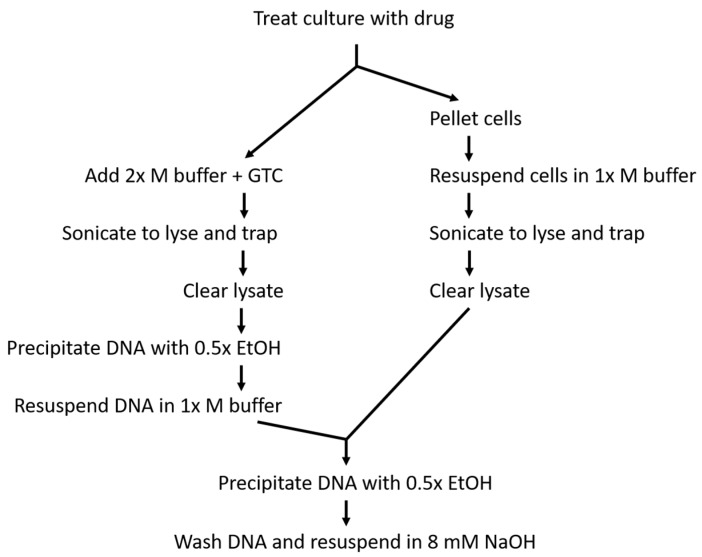
Flow chart outlining methods 19 (on the left branch) and 16 (on the right branch). Equivalent steps are aligned to provide for easier comparison of the similarities and differences in the two protocols.

Methods 16 and 19 differ from each other primarily in cell handling prior to lysis (Figure 2). In method 16, ciprofloxacin-treated cells were pelleted and then resuspended in drug-containing modified M buffer prior to lysis. This allowed DNA to be easily precipitated from the lysate under the same conditions used in the original RADAR assay that prevented simultaneous free protein precipitation. While the original RADAR assay reported a number of conditions that specifically precipitated DNA in the absence of free protein, including the use of proprietary reagents RLTplus and DNAzol, non-proprietary M buffer resulted in the highest yield of covalent complexes [36]. Thus, the more affordable and higher-yielding M buffer (in its modified form [37]) was favored here for DNA precipitation in the bacterial RADAR assay. Although method 16 maintained reliable DNA precipitation conditions, this method also required an additional manipulation step prior to lysis, which subjected the cultures to temperature fluctuation due to the lack of a centrifuge that can easily be maintained at 37 °C. To minimize the fluctuation, the centrifuge was run prior to use to increase the internal temperature.

In method 19, temperature fluctuation prior to lysis was limited due to the lack of a centrifugation step. However, this meant that Luria-Bertani (LB) media would be present in the modified M buffer and could impact what precipitated with the DNA due to the altered pH and ionic strength. Thus, after the initial DNA precipitation and pelleting, the DNA was resuspended in modified M buffer to authentically recreate the precipitation conditions in the original RADAR assay and then precipitated a second time. This prolonged the assay, but increased confidence that free protein was not contaminating the DNA. To this point, rpsC (used here as a representative free protein) was regularly detected in samples that underwent only the initial precipitation, while it was not detected in samples that underwent resuspension and reprecipitation (Figure S1, Supplementary Materials)

**Figure 3:**
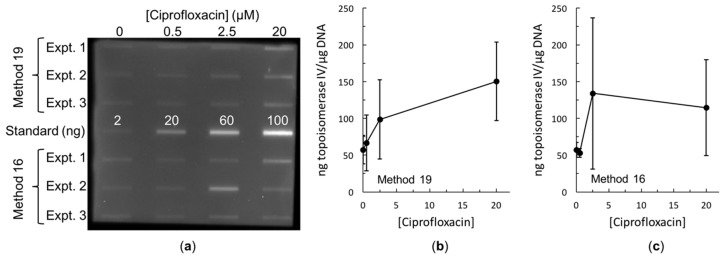
Immunoblot and quantification of ciprofloxacin-induced topoisomerase IV cleavage complexes trapped in *E. coli*. (**a**) Immunoblot comparing methods 16 and 19. Ciprofloxacin concentrations are listed across the top. Three independent experiments of methods 16 and 19 are shown as indicated at the left. In each case, 100 ng of DNA was blotted. To facilitate quantification, 2, 20, 60, and 100 ng of purified *E. coli* topoisomerase IV subunit A were also blotted. (**b**) Quantification of method 19 from (**a**). (**c**) Quantification of method 16 from (**a**). For both (**b**,**c**), a standard curve was generated from the topoisomerase IV standards and used to determine the ng of topoisomerase IV present in each band. This amount was then scaled to determine the number of ng of topoisomerase IV per µg of DNA. Error bars represent the standard deviation of three independent experiments.

**Figure 4:**
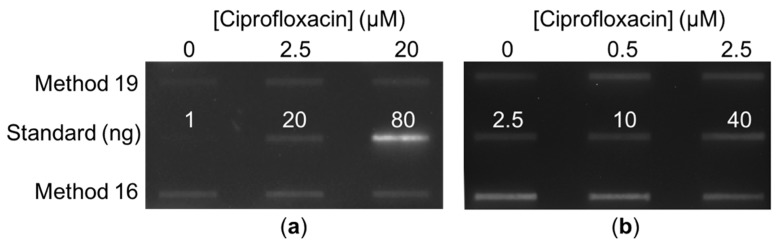
Representative immunoblots of ciprofloxacin-induced type II topoisomerase cleavage complexes trapped in *S. aureus* using either method 16 or method 19 as indicated at the left. (**a**) Immunoblot of ciprofloxacin-induced gyrase cleavage complexes. Purified *S. aureus* gyrase subunit A (1, 20, and 80 ng) was also blotted. (**b**) Immunoblot of ciprofloxacin-induced topoisomerase IV cleavage complexes. Purified *S. aureus* topoisomerase IV subunit A (2.5, 10, and 40 ng) was also blotted. For both (**a**,**b**), ciprofloxacin concentrations present in each sample are indicated at the top. A total of 100 ng of DNA was blotted in each case.

**Figure 5:**
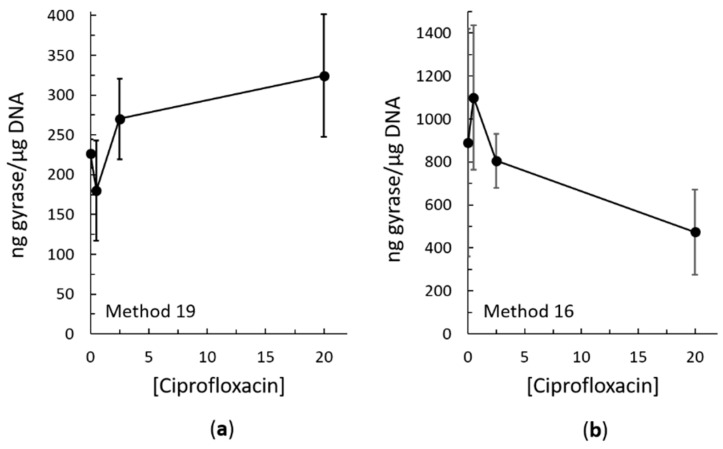
Ciprofloxacin-induced intracellular cleavage complex formation by gyrase in *E. coli* as measured using method 19 (**a**) and method 16 (**b**). Quantification was carried out as described in Figure 3 using a simultaneously blotted purified *E. coli* gyrase subunit A standard. In (**a**), error bars represent the standard error of the mean of two independent experiments. In (**b**), error bars represent the standard deviation of three independent experiments.

Both methods 16 and 19 resulted in trapping of topoisomerase IV cleavage complexes on the DNA in both the Gram-negative species *E. coli* (Figure 3) and the Gram-positive species *Staphylococcus aureus* (Figure 4b). Both methods also resulted in trapping of gyrase cleavage complexes in both the Gram-negative and Gram-positive species (Figure 4a and Figure 5). Because intracellular cleavage complex formation with a different quinolone (norfloxacin) in *E. coli* has been reported but there is no equivalent published data for comparison for *S. aureus*, results with topoisomerase IV and gyrase from *E. coli* were used to judge whether method 19 or method 16 was better. When comparing between methods 16 and 19, quantification revealed that the expected increase in topoisomerase IV cleavage complexes was observed (Figure 3b,c). However, method 19 resulted in a smoother curve that followed the expected trend more so than did method 16. In addition, variability in the amount of cleavage complexes trapped at each drug concentration was reduced in method 19 as compared to method 16. When comparing between methods 16 and 19 with gyrase, the expected increase in intracellular cleavage complexes was observed with method 19 but not with method 16 (Figure 5). Based on these analyses, method 19 appears to give the most consistent results, and therefore, we have dubbed this method the “bacterial RADAR assay.” (The full protocol for this bacterial RADAR assay, referred to above as method 19, can be found in the Supplementary Materials.)

## 3. Discussion

We developed a RADAR-based assay [36] for use in bacteria. This “bacterial RADAR assay” includes the rapid lysis of bacterial cells with simultaneous trapping of topoisomerase IV and gyrase cleavage complexes on the DNA. The rapid lysis step allows cleavage complexes to be trapped before they dissociate, resulting in an accurate intracellular measure of quinolone activity against type II topoisomerases. Thus, this assay can be used to complement mechanistic studies that are carried out in purified systems to confirm their validity and account for confounding factors introduced in the whole-cell context. Examining quinolone activity in the cellular context could provide new mechanistic insight to quinolone action and resistance, and provides an additional tool for examining newly designed topoisomerase-targeted drugs to determine whether they enter bacterial cells and cause cell death by affecting topoisomerase activity. Due to rising rates of quinolone resistance [17,29,44], it is imperative that new antibacterials be developed, and this assay will allow for time-efficient and reagent-efficient additional, meaningful screening of such compounds that are designed to target topoisomerases.

As mentioned above, a bacterial assay based on the ICE assay method was previously developed [35]. Although *E. coli* was the species used, the quinolone norfloxacin (rather than ciprofloxacin) was the drug used to treat the cultures. Thus, it is difficult to make many direct comparisons between their bacterial ICE assay and our bacterial RADAR assay. However, in both cases one sample contained no drug to establish a baseline. With the bacterial ICE assay, ≈0.005 ng topoisomerase IV/µg DNA was reported [35], which is much lower than the ≈57 ng topoisomerase IV/µg DNA found in this work (for both methods 16 and 19; see Figure 3b,c). Similarly, that work found ≈1 ng gyrase/µg DNA [35], which is again much lower than the ≈225 ng gyrase/µg DNA seen above (for method 19; see Figure 5a). One of many possible explanations for this difference is based on the lysis step. Our lysis step took approximately five to ten minutes to complete, depending on whether method 19 or 16 was used, respectively. On the other hand, the bacterial ICE assay utilizes a one-hour lysis step, with a room temperature cell pelleting centrifugation immediately before [35], which is similar to the initial step in our method 16. In vitro, *E. coli* topoisomerase IV cleavage complexes that are stabilized by a drug have a very short half-life [41], and those that form in the absence of a stabilizing drug presumably have a much shorter half-life, as has been seen in other species [37,40,43]. No equivalent in vitro stability data have yet been reported for *E. coli* gyrase. Thus, the difference between the results of the two studies could reflect this instability and dissociation of the complexes during the one-hour lysis. This illustrates why we aimed to determine a rapid lysis method during the development of our bacterial RADAR assay. 

Likewise, due to the suspected high instability of *E. coli* topoisomerase IV cleavage complexes and the affordability of ciprofloxacin, we added an additional aliquot of drug when resuspending the cells in modified M buffer in method 16 and when adding the culture to the “2x” modified M buffer in method 19 so that a constant drug concentration would be maintained until lysis and trapping of cleavage complexes. When method 19 was tested without this additional drug, the difference in the amount of cleavage complexes trapped did not appear to be statistically significant (Figure S2, Supplementary Materials). Thus, the necessity of this additional drug should be determined empirically for each covalent complex being measured, as it may be absolutely necessary for physiologically accurate results for shorter-lived complexes, while dispensable for longer-lived complexes.

In a broader context, the bacterial RADAR assay is easily accessible and could have wider applications outside of the testing of quinolones (and other topoisomerase-targeted compounds) in bacterial cells. The most specialized equipment that it requires is a sonicator, which is much more affordable and smaller than most other common lab tools. Importantly, our assay does not require an ultracentrifuge, which is a large, expensive piece of lab equipment that is often not available at smaller institutions. Furthermore, only small volumes of culture and other reagents are required, thus making this an affordable assay. In addition, the time from culture treatment to blot analysis is less than 48 hours. Presumably, in a similar fashion to the original RADAR assay [36], the bacterial RADAR assay could be used to measure the activity of other proteins that covalently interact with DNA, regardless of whether those proteins are drug targets. 

## 4. Materials and Methods

### 4.1. Strains, Antibodies, and Reagents

*Escherichia coli* and *Staphylococcus aureus* were from Presque Isle Cultures (Erie, PA, USA). A 40 mM ciprofloxacin (Sigma, St. Louis, MO, USA) stock solution was prepared in 0.1 N sodium hydroxide and diluted to 8 mM in 10 mM Tris-HCl (pH 7.9) prior to use to neutralize the base. Subsequent dilutions were also done in 10 mM Tris-HCl (pH 7.9). The primary antibody to *E. coli* and *S. aureus* DNA gyrase subunit A (ab75594) was from AbCam (Cambridge, MA, USA). Polyclonal primary antibodies to *E. coli* topoisomerase IV subunit A and *S. aureus* topoisomerase IV subunit A were produced in rabbit by Thermo Scientific Pierce (Waltham, MA, USA) custom antibody service (90-day protocol) with peptide immunogens KLRPEELQKVTGERGRRG (708:725) and SFIVDTDDFGEVIDMYIS (783:800), respectively. Goat anti-rabbit secondary antibody conjugated to horseradish peroxidase was from AbCam (ab97051). Clarity and ClarityMAX western ECL blotting substrates were from Bio-Rad (Hercules, CA, USA). QuantiFluor dsDNA system was from Promega (Madison, WI, USA).

### 4.2. Culture Growth and Treatment

All cultures were grown in LB broth in a shaking 37 °C water bath. *E. coli* and *S. aureus* cultures (50 mL volume) were inoculated 1:20 from an overnight culture. Cultures were grown to early log phase. Then, aliquots of culture were transferred to warmed tubes for treatment with 0, 0.5, 2.5, or 20 µM ciprofloxacin. These drug concentrations are within the tissue and serum levels observed in the clinic and in animal models [45,46,47] and are in the range tested in purified systems [41]. Drug treatment lasted for 1 h for *E. coli* and 30 min for *S. aureus*.

### 4.3. Cell Lysis and DNA Capture

After drug treatment, equal volumes of culture and warmed “2x” modified M buffer (6 M guanidine thiocyanate (GTC), 40 mM Tris (pH 7.9), 40 mM ethylenediaminetetraacetic acid disodium salt (Na_2_EDTA; pH 8.0), 4% Triton X-100, 2% sarkosyl, 2% dithiothreitol (DTT), 0.2 M sodium acetate (pH 5.2), and sodium hydroxide to bring to pH 6.5) were combined, and additional solid GTC was added and dissolved to give a final concentration of 4 M. (For example, 2 mL of culture mixed with 2 mL of “2x” modified M buffer would require 0.472 g of solid GTC. Due to solubility limits, a concentration of 8 M GTC could not be achieved in the “2x” modified M buffer, and the buffer had to be heated to 37 °C to solubilize all chemicals prior to adjusting the pH). An additional drug aliquot was also added to maintain a constant concentration through the cell lysis process. 

*E. coli* cultures were lysed via sonication of five passes of 15 s each at 60% power. *S. aureus* cultures were lysed by sonication of five passes of 15 s each at 80% power. Large debris was pelleted by centrifuging at 4780× *g* for 5 min. DNA was precipitated as previously described [36] and resuspended in modified M buffer [37] (4 M GTC, 20 mM Tris-HCl (pH 7.9), 20 mM Na_2_EDTA (pH 8.0), 2% Triton X-100, 1% sarkosyl, 1% DTT, 0.1 M sodium acetate (pH 5.2), and sodium hydroxide to bring the pH to 6.5). A second round of DNA precipitation was completed as above. The DNA pellet was then washed and resuspended in 8 mM sodium hydroxide as previously described [36]. 

### 4.4. DNA Quantification and Blotting

DNA concentration was determined using the QuantiFluor dsDNA system per manufacturer’s protocol with the QuantiFluor ST fluorometer (Promega). Prior to blotting, 100 ng of DNA was diluted into 25 mM sodium phosphate (pH 6.5) for a total volume of 100 µL. Samples were blotted on nitrocellulose alongside purified topoisomerase IV or gyrase subunit A from the same species.

Blots were blocked in 5% non-fat dry milk dissolved in TBST (0.02 M Tris, 0.137 M sodium chloride, 0.1% Tween-20, pH 7.6). All washes were done in TBST, and all antibody dilutions were also in TBST. Gyrase blots were incubated with 1:250 primary antibody at room temperature with shaking overnight. Secondary antibody (1:4000) was applied for 5 h. Topoisomerase IV blots were incubated with 1:2000 primary antibody at room temperature with shaking for 3 h. Secondary antibody (1:10,000) was applied for 2 h. Blots were visualized using a CCD camera (Fotodyne FOTO/Analyst Luminary FX) (Hartland, WI, USA) and quantified using AlphaEaseFC 4.0 standalone software (Alpha Innotech) (San Leandro, CA, USA).

## Supplementary Materials

The following are available online at https://www.mdpi.com/2079-6382/8/1/17/s1, Figure S1, Immunoblot for rpsC comparing one vs. two rounds of DNA precipitation. Figure S2, Effects of maintaining a constant drug concentration through cell lysis. Bacterial RADAR Assay Protocol.
