# Supplementary material for: A RADAR-Based Assay to Isolate Covalent DNA Complexes in Bacteria
Source: Antibiotics (Basel). 2019 Feb 27;8(1):17. doi: 10.3390/antibiotics8010017 (PMC6466838; doi:10.3390/antibiotics8010017)
Supplement: Supplementary file 1 [file antibiotics-08-00017-s001.pdf]

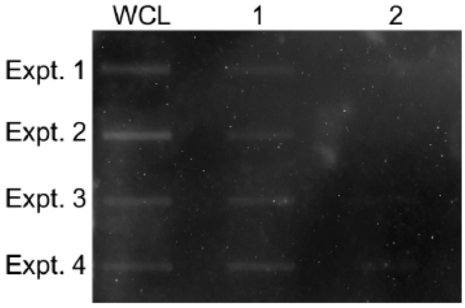

Figure S1. Immunoblot for rpsC comparing one vs two rounds of DNA precipitation. Cleared whole-cell lysate (WCL) acquired using method 19 was precipitated as described and then either washed and resuspended in sodium hydroxide (1) or resuspended in modified M buffer and reprecipitated (2) before being washed and resuspended in sodium hydroxide. The volume of cleared whole-cell lysate that was blotted was approximately equal to the volume from which the 100 ng of DNA that was blotted was acquired from. rpsC primary antibody (22B12B2) was from DSHB and was used at 1:1500. Goat antimouse secondary antibody conjugated to horseradish peroxidase (A4416) was from Sigma and was used at 1:5000.

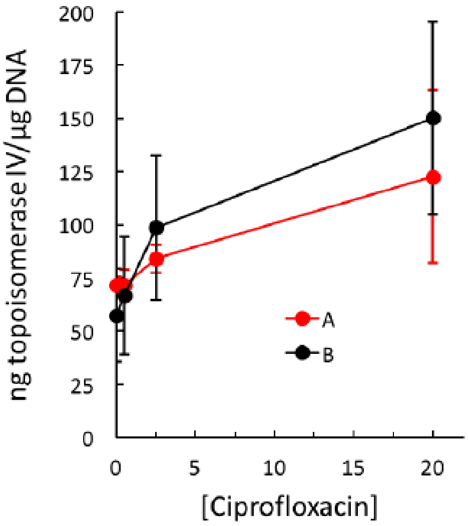

Figure S2. Effects of maintaining a constant drug concentration through cell lysis. Using method 19, additional drug was either not added (A; red circles) or added (B; black circles) once the culture was diluted into "2x" modified M buffer + GTC prior to sonication. Thus, for A (red circles), following the one-hour drug incubation, the drug concentration was diluted by half prior to sonication, while in B (black circles), the additional drug aliquot maintained the one-hour drug incubation concentration through the lysis process. Error bars represent the standard deviation of three independent experiments.

Antibiotics **2019**, *8*, 17; doi:10.3390/antibiotics8010017

Bacterial RADAR Assay Protocol

Note that this is "Method 19" from the article. Blank lines are inserted to indicate necessary or convenient daily endpoints.

1. Grow overnight culture of desired bacteria.

2. Seed 50 mL culture at a 1:20 dilution, then grow until early log phase (OD600 ≈0.3). Place solutions and vessels in 37 °C waterbath to warm.
3. Aliquot 3 mL culture to each warmed glass culture tube, treat with drug, and incubate for the desired length of time.
4. Transfer 2 mL treated culture to warmed 15 mL plastic tubes containing 2 mL of "2x" modified M buffer and 0.472 g solid GTC. If desired, add additional drug to maintain the initial drug concentration in light of the additional volume. Vortex briefly to mix and dissolve most of the solid GTC.
5. Sonicate Gram-negative species for 5 × 15 s at 60% power and Gram-positive species for 5 × 15 s at 80%. Place tubes in 37 °C waterbath between sonication passes to act as heat sink and maintain temperature.
6. Pellet cell debris at minimum 4780 × *g* for 5 min.
7. Transfer DNA-containing supernatant to glass tube for precipitation with a 0.5 volume of 100% ethanol. Vortex, then precipitate on ice for 1 h.
8. Pellet DNA in 1.5-mL tubes (use multiple spins if necessary to acquire DNA from desired lysate volume) at 13,400 × *g* for 20 min at room temperature.
9. Remove supernatant, and resuspend DNA pellet in 800 μL of modified M buffer.
10. Add 0.5 volume 100% ethanol, vortex, and precipitate on ice for 1 h.
11. Pellet DNA at 13,400 × *g* for 20 min at room temperature.
12. Wash DNA twice: add 1 mL 75% ethanol, vortex, and pellet as above for 10 min.
13. Dry DNA pellet, then resuspend in 100 μL of freshly diluted 8 mM NaOH.

14. Determine DNA concentration using the QuantiFluor dsDNA system (Promega) or equivalent that measures double-stranded DNA concentration.
15. Dilute 100 ng DNA (or desired amount) into 25 mM NaP (pH 6.5) to a total volume of 100 μL.
16. Blot samples on 0.45 nm nitrocellulose membrane pre-soaked in 25 mM NaP (pH 6.5).
17. Rinse membrane three times with TBST, and wash for 15 min in TBST.
18. Block membrane for one hour or overnight in 5% non-fat dry milk in TBST.
19. Rinse membrane three times with TBST, then wash for 15 min and then 2 × 5 min with TBST.
20. Incubate with primary antibody for 3 h or overnight (depending on antibody being used).
21. Repeat step 19.
22. Incubate with secondary antibody for 2 h.
23. Repeat step 19 with one additional final 5-min wash.
24. Visualize using ECL western blotting substrate and CCD camera. Exposures may need to range from 15 s to 10 min depending on the antibodies used, amount of DNA blotted, and amount of target bound to the DNA.

Notes:

1. "2x" modified M buffer consists of 6 M guanidine thiocyanate (GTC), 40 mM Tris (pH 7.9), 40 mM Na_2EDTA (pH 8.0), 4% Triton X-100, 2% sarkosyl, 2% DTT, 0.2 M sodium acetate (pH 5.2), and sodium hydroxide to bring the pH to 6.5. Do not add strong acid to this solution as harmful gases can be produced and released. This solution must be prepared at a minimum of 37°C for solubility purposes. It can be stored at room temperature and then reheated to re-dissolve all components. Additional solid GTC is added to individual sonication tubes at the time of use so that the final GTC concentration prior to sonication is 4 M.

2. Modified M buffer consists of 4 M guanidine thiocyanate (GTC), 20 mM Tris-HCl (pH 7.9), 20 mM Na_2EDTA (pH 8.0), 2% Triton X-100, 1% sarkosyl, 1% DTT, 0.1 M sodium acetate (pH 5.2), and sodium hydroxide to bring the pH to 6.5.
3. TBST consists of 0.02 M Tris, 0.137 M sodium chloride, and 0.1% Tween-20, brought to pH 7.6.
4. Volumes of culture treated and used in DNA precipitation can be scaled according to the desired DNA yield, which may require resuspending the final DNA pellet in a larger volume.
5. Sonication powers are based on using the Qsonica Q125 with 1/8" tip.
6. GTC is hazardous waste. Thus, discarded M buffer-containing solutions should be handled accordingly.
